# Genome-wide DNA methylation and gene expression patterns reflect genetic ancestry and environmental differences across the Indonesian archipelago

**DOI:** 10.1371/journal.pgen.1008749

**Published:** 2020-05-26

**Authors:** Heini M. Natri, Katalina S. Bobowik, Pradiptajati Kusuma, Chelzie Crenna Darusallam, Guy S. Jacobs, Georgi Hudjashov, J. Stephen Lansing, Herawati Sudoyo, Nicholas E. Banovich, Murray P. Cox, Irene Gallego Romero

**Affiliations:** 1 Center for Evolution and Medicine, School of Life Sciences, Arizona State University, Tempe, Arizona, United States of America; 2 The Translational Genomics Research Institute, Phoenix, Arizona, United States of America; 3 Melbourne Integrative Genomics, University of Melbourne, Parkville, Australia; 4 School of BioSciences, University of Melbourne, Parkville, Australia; 5 Centre for Stem Cell Systems, University of Melbourne, Parkville, Australia; 6 Genome Diversity and Diseases Laboratory, Eijkman Institute for Molecular Biology, Jakarta, Indonesia; 7 Complexity Institute, Nanyang Technological University, Singapore, Singapore; 8 Statistics and Bioinformatics Group, School of Fundamental Sciences, Massey University, Palmerston North, New Zealand; 9 Santa Fe Institute, Santa Fe, New Mexico, United States of America; 10 Vienna Complexity Science Hub, Vienna, Austria; 11 Stockholm Resilience Center, Kräftriket, Stockholm, Sweden; 12 Department of Medical Biology, Faculty of Medicine, University of Indonesia, Jakarta, Indonesia; 13 Sydney Medical School, University of Sydney, Sydney, NSW, Australia; New York Genome Center & Columbia University, UNITED STATES

## Abstract

Indonesia is the world’s fourth most populous country, host to striking levels of human diversity, regional patterns of admixture, and varying degrees of introgression from both Neanderthals and Denisovans. However, it has been largely excluded from the human genomics sequencing boom of the last decade. To serve as a benchmark dataset of molecular phenotypes across the region, we generated genome-wide CpG methylation and gene expression measurements in over 100 individuals from three locations that capture the major genomic and geographical axes of diversity across the Indonesian archipelago. Investigating between- and within-island differences, we find up to 10.55% of tested genes are differentially expressed between the islands of Sumba and New Guinea. Variation in gene expression is closely associated with DNA methylation, with expression levels of 9.80% of genes correlating with nearby promoter CpG methylation, and many of these genes being differentially expressed between islands. Genes identified in our differential expression and methylation analyses are enriched in pathways involved in immunity, highlighting Indonesia's tropical role as a source of infectious disease diversity and the strong selective pressures these diseases have exerted on humans. Finally, we identify robust within-island variation in DNA methylation and gene expression, likely driven by fine-scale environmental differences across sampling sites. Together, these results strongly suggest complex relationships between DNA methylation, transcription, archaic hominin introgression and immunity, all jointly shaped by the environment. This has implications for the application of genomic medicine, both in critically understudied Indonesia and globally, and will allow a better understanding of the interacting roles of genomic and environmental factors shaping molecular and complex phenotypes.

## Author summary

Understanding how gene expression varies across individuals and environments is fundamental for the success of molecular medicine. However, the vast majority of studies of gene expression, particularly in healthy individuals, have primarily involved populations of European descent. It is unclear whether these findings are generalizable to the rest of humanity. We examined gene expression and DNA methylation in 116 individuals across Indonesia, the world's fourth most populous country. Our three populations–Mentawai off the western coast of Sumatra, Sumba in central Indonesia, and a group of hunter-gatherers, the Korowai, living in the province of Indonesian New Guinea–span the main axes of genetic and geographic diversity in the region. Up to 10% of genes show differences in expression and methylation patterns between islands, with many involved in immune function. Variation between villages within each location can likely be attributed to small-scale environmental differences. These findings emphasise the need to consider a broad range of genetic and environmental backgrounds when examining how molecular patterns and processes are shared across human populations.

## Introduction

Modern human genomics does not equitably represent the full breadth of humanity. While genome sequences for people of European descent now number a million or more, most of the world is deeply understudied [1]. This is particularly true of Indonesia [2], a country geographically as large as continental Europe and the world’s fourth largest by population. Genomic diversity in Indonesia is strikingly different to other well-characterized East Asian populations, such as Han Chinese and Japanese, but this diversity is not captured in large global datasets like the 1000 Genomes Project [3] or the Simons Genome Diversity Project [4]. The first three Indonesian genome sequences were only reported in 2016 [5] with the first representative survey of diversity across the archipelago only appearing in 2019 [6]. This extreme lack of representation extends to molecular phenotypes. To our knowledge, only one genome-wide gene expression study has been published [7] from the region, focused exclusively on host-pathogen interactions with *P*. *falciparum*. There are no analyses of diversity in gene regulatory mechanisms in either Indonesia or, more broadly, Island Southeast Asia.

This gap is especially incongruous because Indonesia is an epicenter of infectious disease diversity, ranging from well-known agents like malaria [8] to emerging diseases like zika virus [9]. The country faces substantial healthcare challenges, including the rise in prevalence of understudied tropical infectious diseases and the increasing impact of metabolic disorders among a growing middle class [10]. However, Indonesia also offers unique advantages for studying responses to these diseases and disorders, some of which are likely to have exerted strong evolutionary pressures on the immune system over thousands of years [11]. Because the country comprises a chain of islands that stretch for 50 degrees of longitude along the equator (wider than either the continental USA or mainland Europe), but span barely 15 degrees of latitude, environment conditions are broadly comparable in many key respects across Indonesia. In contrast, a complex population history means that its people differ greatly, forming a genomic cline of Asian ancestry in the west to Papuan ancestry in the east [12]. This change in ancestry is the most distinctive genomic signal observed in the region [13], and, since Papuans derive up to 5% of their genomes from Denisovans, also gives rise to an east-west gradient of archaic introgression [6]. Altogether, the unique conditions observed in Indonesia provide a framework for studying the effects of genome composition on gene expression in a heterogeneous environment.

To provide a benchmark dataset of regional molecular phenotypes, here we report genome-wide measurements of DNA methylation and gene expression for 116 individuals drawn from three population groups that capture the major genomic and geographical axes of diversity across Indonesia. The people of Mentawai, living on the barrier islands off Sumatra, are representative of the dominant Asian ancestry in western Indonesia [13]; the Korowai, hunter-gatherers from the highlands of western New Guinea island capture key aspects of regional Papuan ancestry [6]; and the inhabitants of Sumba in eastern Indonesia are, genetically, a near equal mixture of the two different ancestries [14]. However, it remains unclear whether, and to what extent, these differences in genetic ancestry correlate with variation in molecular phenotypes. By quantifying DNA methylation and gene expression levels across Indonesia for the first time, we identify the relative influences of genomic ancestry versus plasticity to local environmental conditions in driving regional molecular phenotypic patterns.

## Methods

### Ethics statement

The samples used in this study were collected by HS, JSL and an Indonesian team from the Eijkman Institute for Molecular Biology, Jakarta, Indonesia, with the assistance of Indonesian Public Health clinic staff. All collections followed protocols for the protection of human subjects established by institutional review boards at the Eijkman Institute (EIREC #90 and EIREC #126) and the University of Melbourne (Human Ethics Sub-Committee approval 1851639.1). All individuals gave written informed consent for participation in the study. Permission to conduct research in Indonesia was granted by the Indonesian Institute of Sciences and by the Ministry for Research, Technology and Higher Education.

### Data collection

Whole blood was collected by trained phlebotomists from the Eijkman Institute and local community health centers from over 300 Indonesian men. Samples were collected across multiple villages in the three islands using EDTA blood tubes from either Vacuette or Intherma for DNA isolation, and Tempus Blood RNA Tubes (Applied Biosystems) for RNA isolation. Samples were collected in 2016 in the course of three distinct field trips: Korowai samples were collected in February, Mentawai samples in April, and Sumba samples in July. RNA extractions were performed according to the manufacturers’ protocols after all collections had taken place and randomised with respect to village and island (S1 and S2 Tables).

Quality and concentration of all extracted RNA samples were assessed with a Bioanalyzer 2100 (Agilent) and a Qubit device (Life Technologies), respectively. We selected 116 male samples for RNA sequencing and DNA methylation analysis primarily on the basis of their RIN (RNA Integrity Number), by focusing on villages with at least 10 samples with RIN ≥ 6 (Table 1). Given our past work on the island of Sumba [14], we included all samples from Sumba with RIN ≥ 6, heedless of village. However, we occasionally observed differences between our RIN measurements and those performed by our sequencing provider, with the latter generally being lower. Out of 116 individuals, 24 (21%) had a final RIN measurement < 6. Further detail on all samples, including extraction and sequencing batches, is provided in S1 and S2 Tables. Library preparation was performed by Macrogen (South Korea), using 750 ng of RNA and the Globin-Zero Gold rRNA Removal Kit (Illumina) according to the manufacturer's instructions. Samples were sequenced using a 100-bp paired-end configuration on an Illumina HiSeq 2500 to an average depth of 30 million read pairs per individual, in three batches. All batches included at least one inter-batch control for downstream normalisation (S1 and S2 Tables).

**Table 1:** Numbers of DNA methylation and RNA sequenced samples from each study location.

Island	Village	Location	DNA methylation	RNA-seq	RNA-seq samples RIN ≥ 6
Mentawai	Madobag	1.594° S, 99.084° E	17	17	15
	Taileleu	1.788° S, 99.137° E	31	31	31
	*Subtotal*		48	48	46
Sumba	Anakalang	9.588° S, 119.575° E	17	17	15
	Bukambero	9.450° S, 119.104° E	1	1	0
	Hupu Mada	9.697° S, 119.464° E	5	5	0
	Padira Tana	9.671° S, 119.832° E	3	3	2
	Patiala Bawa	9.751° S, 119.332° E	1	1	0
	Rindi	9.935° S, 120.669° E	5	5	2
	Wunga	9.385° S, 119.958° E	16	16	12
	Wura Homba	9.560° S, 118.959° E	1	1	0
	*Subtotal*		49	49	39
New Guinea island	Basman (Korowai)	5.480° S, 139.673° E	19	19	15
	*Subtotal*		19	19	15
Total			116	116	92

In parallel, we extracted whole blood DNA from all individuals included in the RNA sequencing data using Gentra Puregene for human whole blood kit (QIAGEN) and MagAttract HMW DNA kit (QIAGEN) according to the manufacturer's instructions. 1 μg of DNA from each sample was shipped to Macrogen, bisulfite-converted and hybridized to Illumina EPIC BeadChips according to the manufacturer's instructions. Samples were randomised with respect to village and island across two array batches, with three samples processed on both batches to control for technical variation (S1 Table).

### RNA sequencing data processing

All RNA sequencing reads were examined with FastQC v. 0.11.5 [15]. Leading and trailing bases below a Phred score of 20 were removed using Trimmomatic v. 0.36 [16]. Reads were then aligned to the human genome (GRCh38 Ensembl release 90: August 2017) with STAR v. 2.5.3a [17] and a two-pass alignment mode; this resulted in a mean of ~29 million uniquely-mapped read pairs per sample. Next, we performed read quantification with featureCounts v. 1.5.3 [18] against a subset of GENCODE basic (release 27) annotations that included only transcripts with support levels 1–3, retaining a total of 58,391 transcripts across 29,614 genes. On average, we successfully assigned ~15 million read pairs to each sample (S2 Table).

### Variant calling and ancestry estimates

We applied GATK RNA-seq Best Practices [19–21] (https://software.broadinstitute.org/gatk /documentation/article.php?id = 3891) to the mapped RNA-seq data in order to produce a set of genotype variants from each sample and confirm their ancestry. We marked duplicate mapped reads with Picard (http://broadinstitute.github.io/picard) and recalibrated base quality scores against files provided in the GATK Resource Bundle. Variants were called by first producing per-sample raw genotype-likelihoods using HaplotypeCaller, and then joint genotyping all the per-sample gVCFs using GenotypeGVCFs [20,22]. This produced 431,808 variants, from which only biallelic SNPs with <1% missing genotypes were retained. This set was further LD pruned with PLINK v1.90 [23] using a sliding window approach (window size 100 SNPs, step size 10 SNPs, r^2^ threshold 0.2); 180,715 variants passed LD pruning and were further used in principal component and Admixture analyses. All PCAs were performed in PLINK; admixture analyses were carried out using ADMIXTURE v1.3.0 [24] and setting K = 2, 3 or 5. Papuan and Asian ancestry proportions for each sample were estimated using ADMIXTURE results at K = 2, as in [25].

To explore the placement of our samples within a broader geographical context, we also merged our newly generated data with a previously generated genotyping dataset [13] spanning populations sampled across Island Southeast Asia, Papua and Polynesia; including additional samples from Mentawai, Sumba and multiple New Guinean groups. Original genotyping data (roughly 540,000 autosomal SNPs) were translated into hg38 genomic coordinates and merged with the unfiltered RNA-seq data call set. We removed A/T, G/C and triallelic variants from both datasets to avoid strand bias prior to merging, and applied a 5% missingness filter to the merged dataset. This produced 13,233 overlapping SNPs which were analysed by PCA in PLINK as above.

We elected not to directly infer Denisovan introgression on this call set due to its non-random missingness relative to whole-genome sequencing data, and the high likelihood that differences in gene and exon length would impact our ability to identify introgression in an unbiased way across all expressed genes.

### Deconvolution of blood cell type proportions

Because blood cell type composition can impact gene expression estimates in bulk RNA samples, we used DeconCell v. 0.1.023 [26] to estimate the proportion of CD8T, CD4T, NK, B cells, monocytes and granulocytes in each sample (S2 Table), and tested these for association with the first 10 PCs of both the methylation and expression datasets. Unfiltered read counts were normalised using the inbuilt Decon-cell command ‘dCell.expProcessing’, which performs TMM normalization, log_2_ transformation of the counts, and then scale normalization for each gene. The proportion of each cell type was then predicted using the normalized data and the reference bulk dataset. In addition, we also tested the methylation-based approach from Houseman et al [27], as well as an additional RNA-seq based method, ABIS [28]. Overall, we observed high similarity between all three methods (S3 Table), especially between Decon-cell and the Houseman et al method, with Pearson's R for individual cell types ranging between 0.47 in CD8T cells to 0.81 in B cells. However, we found that the methylation-based approach yielded erratic variations in the fraction of different cell types, even between methylation replicates. We therefore decided to use DeconCell, which more closely mirrored the proportions of cell types found in healthy samples. Further details on the blood deconvolution are available as S1 Text.

### Differential expression analysis

All statistical analyses were performed using R v. 3.5.2 [29]. We transformed read counts to log_2_-counts per million (CPM) using a prior count of 0.25 and removed genes with low expression levels by only keeping genes with log_2_ CPM ≥ 1 in at least half of the individuals from any island, resulting in a total of 13,031 genes retained for further analysis. To quantify the effect of technical batches, we included six replicate samples among our sequencing batches. As anticipated, PCA of uncorrected data suggested the presence of substantial sequencing batch effects in the data (S1 Fig). However, pairwise correlations between technical replicates were higher than between different individuals from the same village sequenced in the same batch (S2 Fig).

We applied TMM normalisation [30] to the data, and removed high sample variability from the count data using the *voom* function [31] in limma v. 3.40.2 [32]. Differential expression testing was also performed using limma. To construct the linear model for testing, we used ANOVA to test for associations between all possible covariates and the first 10 principal components (PC) of the data. Technical covariates significantly associated with at least one PC (sequencing batch, RIN, age) were included in the differential expression testing model. Sampling sites were included at either the island or the village level, depending on the test. Comparisons between villages were limited to those with at least 15 individuals, to ensure sufficient power to detect differences. All individuals were included in comparisons between islands, and models were not hierarchically structured. Genes were called as differentially expressed (DEG) if the FDR-adjusted *p* value was below 0.01, regardless of the magnitude of the log_2_ fold change, unless noted otherwise.

Lists of DEGs were annotated using biomaRt v. 2.40.0 [33]. Gene set enrichment analyses for the DEGs on the island and village levels were performed using clusterProfiler v. 3.12.0 [34], with Gene Ontology and KEGG annotation drawn from the org.Hs.eg.db v. 3.9 database [35]. Additionally, we tested whether DEGs were enriched for genes known to have been introgressed from Denisovans into individuals of Papuan ancestry at high frequency using a hypergeometric test. Finally, to examine possible associations between known climatic variables and expression across sampling sites, we retrieved mean monthly precipitation and temperature data from WorldClim v. 2.0 [36] for the five main villages in our study at a resolution of 0.5 arcminutes (roughly 1 km^2^ tiles).

### DNA methylation array data processing and analysis

DNA methylation data were processed using minfi v. 1.30.0 [37]. The two arrays were combined using the ‘combineArrays’ function and preprocessed with the ‘bgcorrect.illumina’ function to correct for array background signal. Signal strength across all probes was evaluated using the ‘detectionP’ function and probes with signal *p* < 0.01 in >75% of samples were retained. To avoid potential spurious signals due to differences in probe hybridization affinity, we discarded 6,072 probes overlapping known SNPs segregating in any of the study populations based on previously published genotype data [6]. The final number of probes retained was 859,404. Subset-quantile Within Array Normalization (SWAN) was carried out using the ‘preprocessSWAN’ function [38]. Methylated and unmethylated signals were quantile normalized using lumi v. 2.36.0 [39]. As with the RNA sequencing, replicate samples were included to detect and correct for batch effects (S3 Fig). The replicate samples exhibit a high correlation between batches (Spearman’s ρ 0.969 for MPI-025 and 0.980 for SMB-ANK-029, S4 Fig). As above, we used limma to test for differential methylation between sampling sites. We included methylation array batch, age, and the estimated cell type proportions (derived from the RNA sequencing data) as covariates. Differentially methylated probes (DMPs) between all pairwise comparisons of the islands and villages were identified using contrast designs. Significant DMPs were selected based on an FDR-adjusted *p* value threshold of 0.01 and a log_2_ fold change of 0.5 or greater. Enrichment tests for the DMPs were performed using missMethyl v. 1.18.0 [40], which accounts for differences in probe density associated with gene length that can otherwise bias results [41]; probes were annotated to genes according to Illumina's manifest for the EPIC array. Significantly enriched pathways were selected based on an FDR-adjusted *p* value of 0.01. In addition, we intersected DMPs with published Epigenome-wide Association Studies available on the EWAS catalogue (http://ewascatalog.org) on November 2019. Altogether, we tested over 100 traits in the catalogue measured in whole blood using the ‘enricher’ universal enrichment analysis tool in clusterProfiler with FDR correction for multiple tests. For each population comparison, we selected methylated CpG sites that have mean beta difference > |0.05| and adjusted *p* < 0.01 against the background methylated CpG sites.

We further identified differentially methylated regions (DMRs) by annotating the CpG probes with the ‘cpg.annotate’ function of the R package DMRcate v. 3.9 [42], and by collapsing the probes to regions using the ‘dmrcate’ function. Individual probes with an FDR-adjusted *p* value ≤ 0.01 and significant DMRs were selected based on a region beta value of 0.5 or greater.

### Modeling gene expression and CpG methylation values as a function of ancestry proportions

In addition to DE and DM testing between populations, we directly applied linear models of the form ([covariate adjusted gene expression or probe methylation level] ~ Papuan ancestry) to each gene or probe to directly assess gene expression and CpG methylation levels as a function of Papuan ancestry proportions (determined through ADMIXTURE analyses as described above). Similarly to other analyses, batch, age, and blood cell type, as well as RIN for gene expression data, were accounted for. We tested for the enrichment of the CpGs and genes identified here among the DMPs and DEGs identified in contrast analyses with Fisher’s exact test.

### Principal Component Analysis of expression and methylation data (PCA)

**Fig 1:**
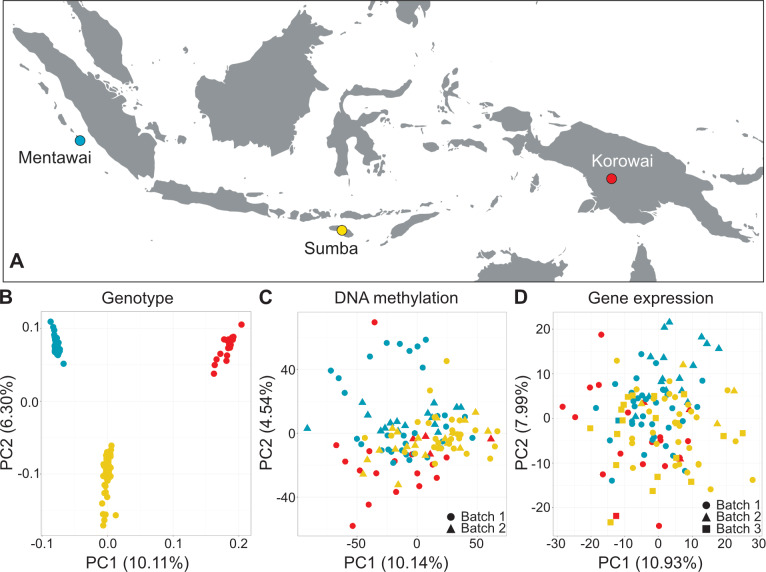
Sampling locations and overview of DNA methylation and gene expression variation among the study samples. (A) Colors indicate island populations: Mentawai, blue; Sumba, yellow; Korowai, red. PCA was performed on the top 10,000 most variable methylation probes and the top 1,000 most variable genes, determined by the sample-wide coefficient of variation. The first two axes of variation from the principal component analysis in the (B) RNA-seq-derived genotype data, (C) DNA methylation and (D) gene expression data after correcting for confounding effects are driven by between-island differences. Plotting shapes indicates sequencing/array batches.

DNA methylation M-values and gene expression log_2_ CPM values were adjusted to correct for batch effects and differences in blood cell type proportions between samples by fitting a linear model with the technical covariates used in the differential methylation and expression analysis. Residuals of this model were used in the PCAs in Fig 1. Variable CpG probes and genes were identified based on coefficients of variation between samples. PCA was performed using the 10^4^ most variable probes and the 10^3^ most variable genes from the methylation and expression datasets, respectively; PCAs of the entire data set before and after batch correction are available in S1 and S3 Figs.

### Identifying associations between DNA methylation regions and gene expression

We used the R package MethylMix v. 2.12.0 [43,44] to identify transcriptionally predictive methylation states by focusing on methylation changes that are associated with gene expression levels. As with the PCA analysis, DNA methylation M-values and gene expression log (CPM) values were adjusted to account for technical covariates and blood cell type proportions by fitting a linear model. Residuals of these linear models were used in the analysis. Batch corrected M-values and logCPM values were min-max normalized to range from 0 to 1. CpG probe methylation levels were matched to genes using the *ClusterProbes* function, which uses a complete linkage hierarchical clustering algorithm for all probes of a single gene to cluster the probes. To identify transcriptionally predictive DNA methylation events, MethylMix utilizes linear regression to detect negative correlations between methylation and gene expression levels. Matching DNA methylation and gene expression data from 116 individuals were used in the analysis, and a total of 10,420 genes with matching methylation and expression data were tested. As MethylMix does not output detailed summary statistics of the fitted linear models, we used linear regression to calculate the r^2^ and *p* values for each significant CpG probe cluster and gene pair detected by MethylMix. False discovery rate adjusted *p* values were calculated using the ‘p.adjust’ function in base R.

## Results

### Differential DNA methylation and gene expression between Indonesian island populations

To quantify the gene regulatory landscape in Indonesia, we generated DNA methylation (array) and gene expression (RNA sequencing) measurements from 116 whole blood samples of male individuals living on three islands in the Indonesian archipelago (Fig 1A). Our three sampling sites, Mentawai, Sumba, and New Guinea, represent distinct points along a well documented Asian/Papuan admixture cline [13]: the Korowai of New Guinea exhibit high Papuan ancestry; Sumbanese have intermediate degrees of Papuan ancestry; and the Mentawai have no Papuan ancestry, having been settled primarily by ancestral Austronesian speakers. Furthermore, Korowai individuals are likely to carry up to 5% of introgressed genomic sequence from archaic Denisovans, as repeatedly observed in other samples from the island of New Guinea [6,45].

Principal component analysis of genotype variants called from the RNA sequencing data shows clear clustering of samples driven by population origin (Fig 1B), demonstrating that the three populations are genetically distinguishable. Similarly, Admixture analyses at K = 3 and K = 5 (S5 Fig) confirm that the three islands represent distinct populations with very limited gene flow between them, alongside a lack of additional fine-scale geographic structure within either Sumba or Mentawai that could confound our analyses despite the inclusion of multiple villages in our sampling strategy. In addition, when analyzed together with 513 samples drawn from 20 diverse populations from the broader (Island) Southeast Asia and Papua regions our samples cluster as expected (S6 Fig).

Inter-island differences are severely attenuated in PCAs of DNA methylation (Fig 1C) and gene expression (Fig 1D), although they are still present. After correcting for known technical confounders, PC1 in the DNA methylation data separates the island of Sumba from both the Korowai (FDR-corrected Tukey's HSD *p* = 5.4×10^−4^) and Mentawai (*p* = 6.8×10^−5^); PC2 further differentiates Sumbanese and Mentawai (*p* = 2.6×10^−3^) and additionally separates Mentawai from Korowai (*p* = 1.9×10^−6^). In the gene expression data, Korowai is separated from Sumba (*p* = 9.1×10^−4^) by PC1, whereas PC2 separates Sumba from Mentawai (*p* = 2.4×10^−4^) and Mentawai from Korowai (*p* = 6.3×10^−4^).

**Fig 2:**
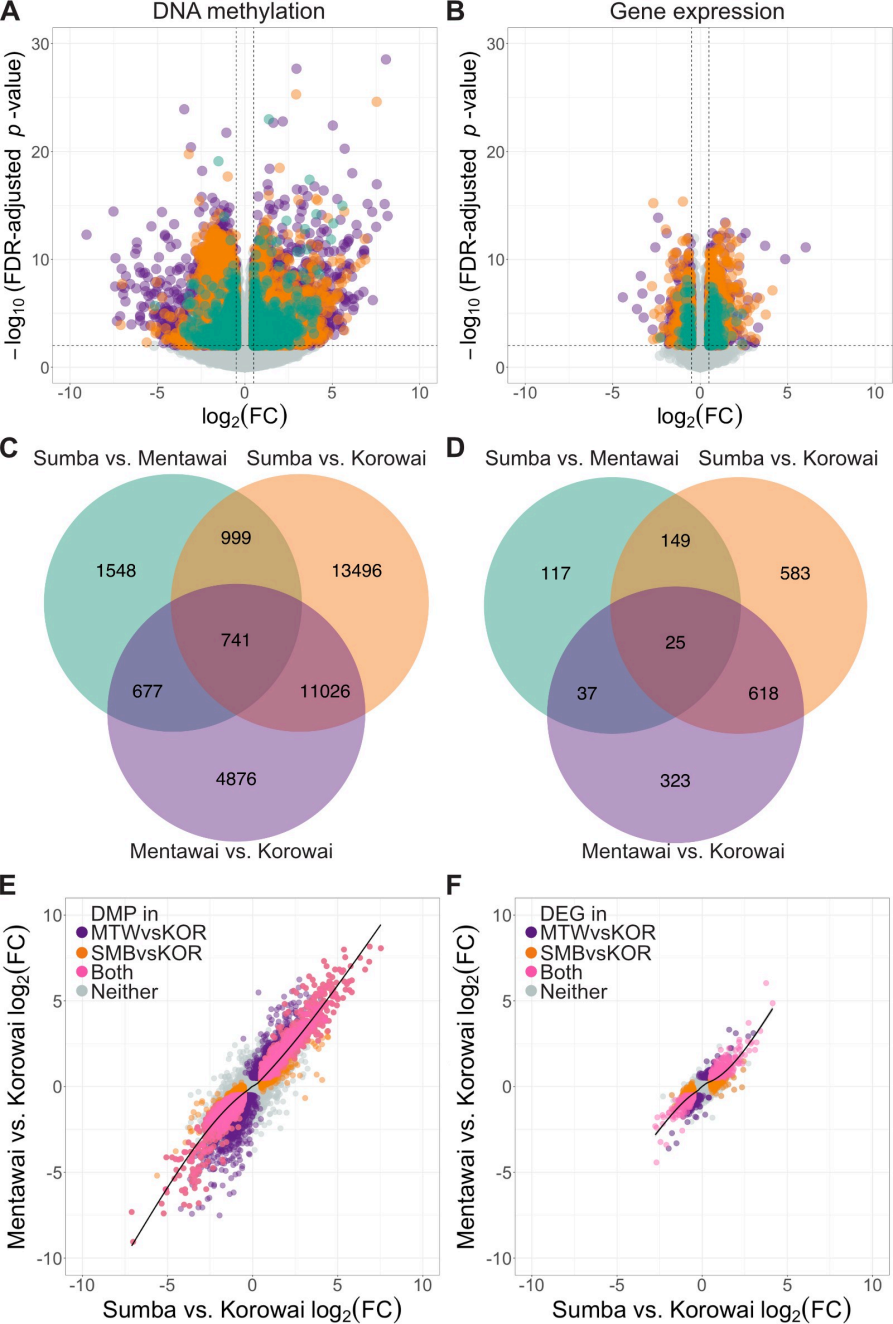
Inter-island differential expression and methylation trends. Volcano plots of (A) differentially methylated probes and (B) differentially expressed genes between Sumba and Mentawai (green), Korowai and Sumba (orange), and Korowai and Mentawai (purple). Venn diagrams of DMPs (C) and DEGs (D) overlapping between different pairwise comparisons at an FDR-adjusted *p* value ≤ 0.01 and an absolute log_2_(FC) ≥ 0.5. Relationship between the log_2_(FC) of each probe (E) and gene (F) between Mentawai vs. Korowai and Sumba vs. Korowai. Probes and genes that were DMP or DEG between Mentawai and Korowai (purple), Sumba and Korowai (orange), or both comparisons (pink) are indicated. Smoothed conditional means based on generalized additive models are presented with 95% confidence intervals.

We then tested for differences in DNA methylation and gene expression between the three islands, initially without considering the village structure in Sumba and Mentawai (Table 1 and S1 and S2 Tables). At an absolute log_2_(FC) threshold of 0.5 and an FDR-adjusted *p* value threshold of 0.01, we detected 26,262 (3.06% of all tested probes), 17,320 (2.02%) and 3,965 (0.46%) differentially methylated probes (DMPs) and 1,375 (10.55% of all tested genes), 1,003 (7.70%), and 328 (2.52%) differentially expressed genes (DEGs) between Sumba and the Korowai, Mentawai and the Korowai, and Sumba and Mentawai, respectively (Fig 2A and 2B). In addition, we identified 1,454, 1,168, and 279 differentially methylated regions across all three inter-island comparisons, respectively, when thresholding to a mean β difference of 0.05 across the region. A full summary of these results is available as S4 Table. We also directly modeled CpG methylation and gene expression levels as a function of the proportion of Papuan ancestry in each sample, identifying 9,305 CpGs and 2,025 genes with an adjusted *p* value <0.01 after correcting for multiple testing (S5 and S6 Tables). These genes and CpGs associated with Papuan ancestry are enriched for DE genes (Fisher’s exact *p* = 5.7 × 10^−125^) and DMPs (*p* <2.2×10^−16^) between islands, suggesting differences in Papuan ancestry levels may be directly driving some of the observed gene regulatory changes in our inter-island comparisons. In particular, when testing for the overrepresentation of these CpGs and genes among the DMPs and DEGs in each of the three pairwise comparisons separately, we find overlaps of 508/1,003 DEGs (50.65%, Fisher’s exact test *p* = 2.4×10^−144^) and 4,927 out of 26,262 DMPs (18.76%, *p* < 2.2×10^−16^) in the comparison between Mentawai (no Papuan ancestry) and the Korowai (100% Papuan), but only 298/1375 DEGs (21.67%, *p* = 1.2×10^−53^) and 1,875/17,320 DMPs (10.83%, *p* < 2.2×10^−16^) between Sumba (roughly 50% Papuan ancestry) and the Korowai.

There is substantial overlap in signals between either Sumba or Mentawai versus Korowai (Fig 2C and 2D). For instance, 44.95% of DEGs between Sumba and Korowai are also differentially expressed between Mentawai and Korowai; the same is true of 41.94% of DMPs between Sumba and Korowai. DEGs and DMPs between Sumba and Mentawai, however, have poor overlap with the other inter-island comparisons and are generally limited in number. This suggests that many of the signals we identify are driven by the Korowai data, and by some degree of homogeneity across Sumba and Mentawai. Indeed, comparisons involving Korowai routinely identify an order of magnitude more DEGs and DMPs. Furthermore, we find substantial agreement in both the magnitude and direction of effect between DEGs and DMPs across both comparisons involving Korowai, (Fig 2E and 2F; the generalized additive model of the form y ~ s(x) was calculated using MGCV with the shrinkage version of the cubic regression spline [46,47]; methylation deviance explained by model = 64.6%, *p* < 2×10^−16^; expression deviance explained = 70.1%, *p* < 2×10^−16^). However, effect size agreement is far poorer when examining both comparisons featuring either Sumba or Mentawai, regardless of whether we focus on methylation or expression differences (S7 Fig).

### Differentially expressed genes are enriched for immune function and Denisovan introgression

We tested for enrichment of DEGs and DMPs against Gene Ontology (GO [48]) and Kyoto Encyclopedia of Genes and Genomes (KEGG [49]) pathways to detect functional enrichment between island populations. Overlapping enriched GO categories and KEGG pathways (adjusted *p* < 0.05; full tables of results for all comparisons are provided in S7−S10 Table) in comparisons between both Mentawai or Sumba versus the Korowai include functions related to the adaptive immune response, malaria response, and nervous system function. However, DEGs between Mentawai and Sumba were not enriched for either GO or KEGG terms. Similar testing for enrichment on DMPs shows various categories, which include terms mostly related to neurogenesis, the nervous system, and immunity, and which partly overlap with categories enriched in DEGs, although the biological interpretation of these results is not straightforward. Thus, to further refine them we intersected our lists of DMPs with published EWAS results available at the EWAS catalogue (http://ewascatalog.org; S11 Table). DMPs associated with a small number of terms are enriched in all three inter-island comparisons; these include immunity associated terms such as HIV infection, as well as lifestyle terms such as smoking behaviour and alcohol consumption, but also less straightforward terms including age.

Finally, because the island of New Guinea has the highest levels of Denisovan introgression worldwide (up to 5% [6]), we asked whether any of the genes differentially expressed between the Korowai (high Papuan ancestry) and Mentawai (no Papuan ancestry), or the Korowai and Sumbanese (intermediate Papuan ancestry) fell within high confidence introgressed Denisovan tracts, on the basis of our previous data [6]. A total of 235 DEGs (considering all comparisons) overlap high confidence introgressed Denisovan haplotype blocks in New Guinea [6]. High-frequency introgressed genes in our DEGs include *FAHD2B* (introgressed at 65% frequency in New Guinea; DE between Sumba and Korowai (*p* = 0.004), and Mentawai and Korowai (*p* = 1.1×10^−6^), and multiple genes related to immunity and antiviral response, such as *CXCR6* (20% frequency in New Guinea [50]) and *GBP1/3/4* (19% frequency in New Guinea [51,52]).

Our ability to identify Denisovan-introgressed genes as differentially expressed depends on both the magnitude of the expression change between the groups being compared, and the introgressed allele’s frequency within our sample. In turn, this means that the proportion of introgressed genes that we expect to be differentially expressed is difficult to predict *a priori*. Therefore, we examined the distribution of introgressed allele frequencies in New Guinea for all DEGs in our data, and asked whether these differ between our three inter-island comparisons. If Denisovan introgression is contributing to expression differences between the three sampling sites, we expect that genes that are differentially expressed between the Korowai and the other two groups will have generally higher introgressed allele frequencies than genes that are DE between the Sumbanese and the Mentawai. Indeed, we observe no difference in allelic frequencies for genes that are DE between both Sumba and Korowai, and Mentawai and Korowai (t-test *p* = 0.902), but observe higher frequencies in DEG between Sumba and Korowai, or Mentawai and Korowai, than between Sumba and Mentawai (*p* = 0.032 and 0.028, respectively), suggesting that Denisovan introgression may impact the expression levels of some genes.

### Methylation changes are correlated with changes in gene expression in a subset of genes

**Fig 3:**
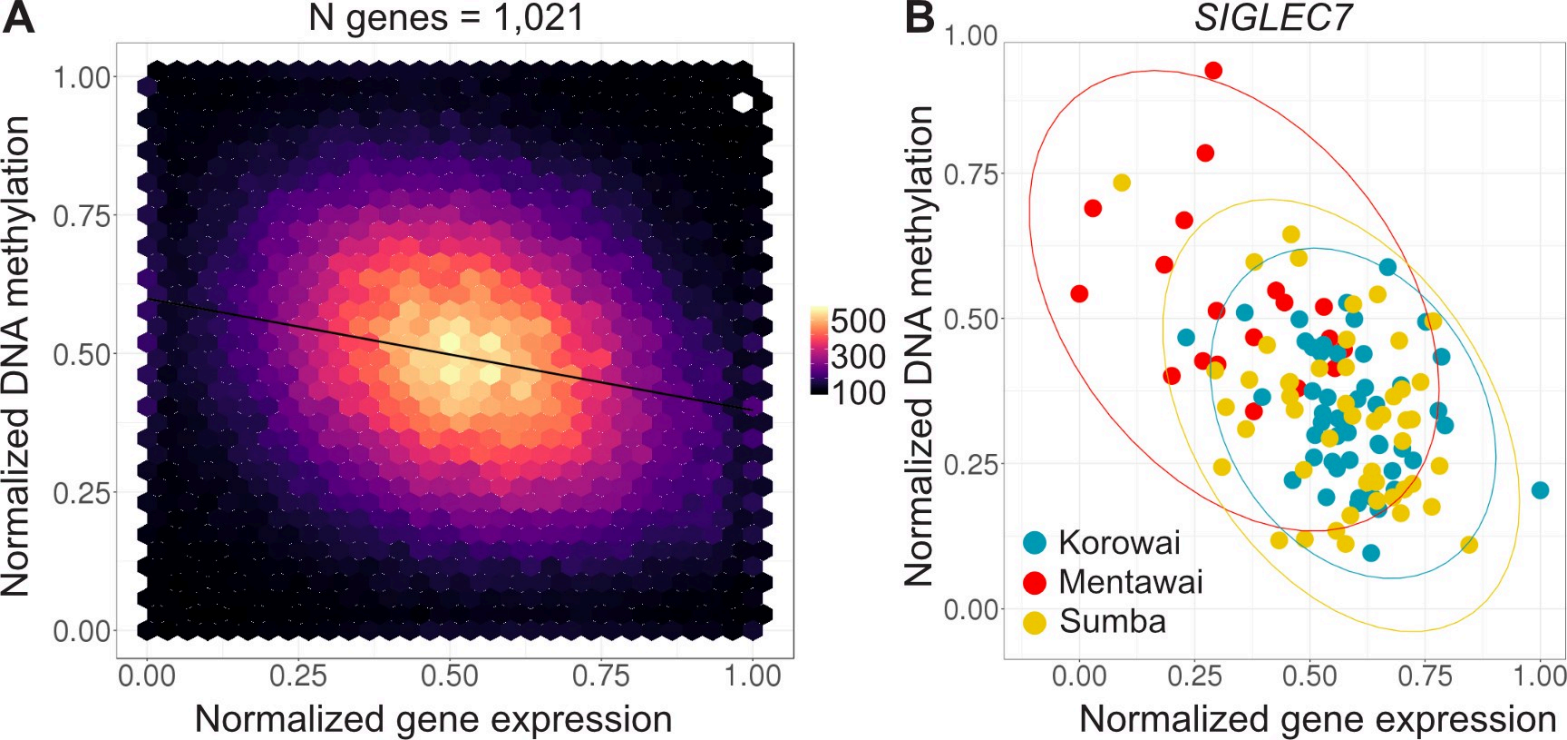
Association between methylation and gene expression levels. (A) Relationship between probe cluster DNA methylation and gene expression levels among the 1,282 probe clusters and associated genes identified by MethylMix. (B) Example of a single gene, *SIGLEC7*, which is both differentially expressed and differentially methylated between Sumbanese and the Korowai.

To further explore the relationship between DNA methylation and gene expression, we asked how much of the variation we observe in gene expression levels can be correlated with variation in DNA methylation levels. We searched for regions of putatively functional DNA methylation by identifying instances of significant negative correlation between gene expression levels and *cis*-promoter methylation. We identified 1,282 probe clusters associated with 1,021 genes (9.80% of all genes with both methylation and expression data) where expression level was associated with nearby CpG methylation (Fig 3A and S12 Table). We compared the genes identified in this analysis with the DMPs and DEGs detected in the between-island comparisons, and find that 218 genes (17.16% of DEGs; hypergeometric *p* = 1.9x10^-14^) in the comparison between Korowai and Sumba, 193 genes (15.20%, *p* = 4.9x10^-22^) between Korowai and Mentawai, and 37 genes (2.91%, *p* = 0.203) between Sumba and Mentawai have expression levels associated with significant methylation changes at nearby CpGs; these include genes like *SIGLEC7* (Fig 3B), which is involved in antigen presentation and natural killer (NK) cell-dependent tumor immunosurveillance [53]. *SIGLEC7* and other *SIGLEC* family genes are also potential immunotherapeutic targets against cancer [54]. There are five enriched KEGG pathways, all broadly involved in immune interactions (S13 Table), including natural killer cell-mediated cytotoxicity. Overall, these results confirm the association between DNA methylation and gene expression and suggest a possible role for differential DNA methylation in shaping the patterns of differential gene expression between these populations.

### Inter-island differences are primarily driven by a subset of villages

While the three island populations differ substantially in terms of genetic composition (Figs 1 and S5), we have previously shown that there is a high degree of genetic similarity within islands [13]. Therefore, we may expect that intra-island differences in either DNA methylation or gene expression profiles, if they exist, are likely to reflect local environmental differences [55]. To test this hypothesis, we took advantage of the fact that we collected samples across multiple villages in both Sumba and Mentawai.

PCA captured differences between villages at both the expression and methylation level (S8 Fig). For instance, PC1 of the DNA methylation data captures varying degrees of separation at both the intra- and inter-island level. Neither the two Sumba villages, Wunga and Anakalang, or the two Mentawai villages, Taileleu and Madobag, are separated by the first PCs, confirming our previous observations of limited differentiation within islands. Between islands, however, PC1 significantly separates multiple pairs of villages, chief amongst them the Korowai from all four other sites (Tukey's HSD-corrected p-values for all comparisons mentioned are available in S14 Table), and three out of four Sumba vs Mentawai village comparisons. There are similar, but again, weaker, trends in the expression data: PC1 separates the Korowai from both Sumba villages, as well as the villages of Wunga (in Sumba) and Madobag (in Mentawai), whereas PC2 separates Taileleu (in Mentawai) from the Korowai, and from Anakalang (in Sumba).

**Fig 4:**
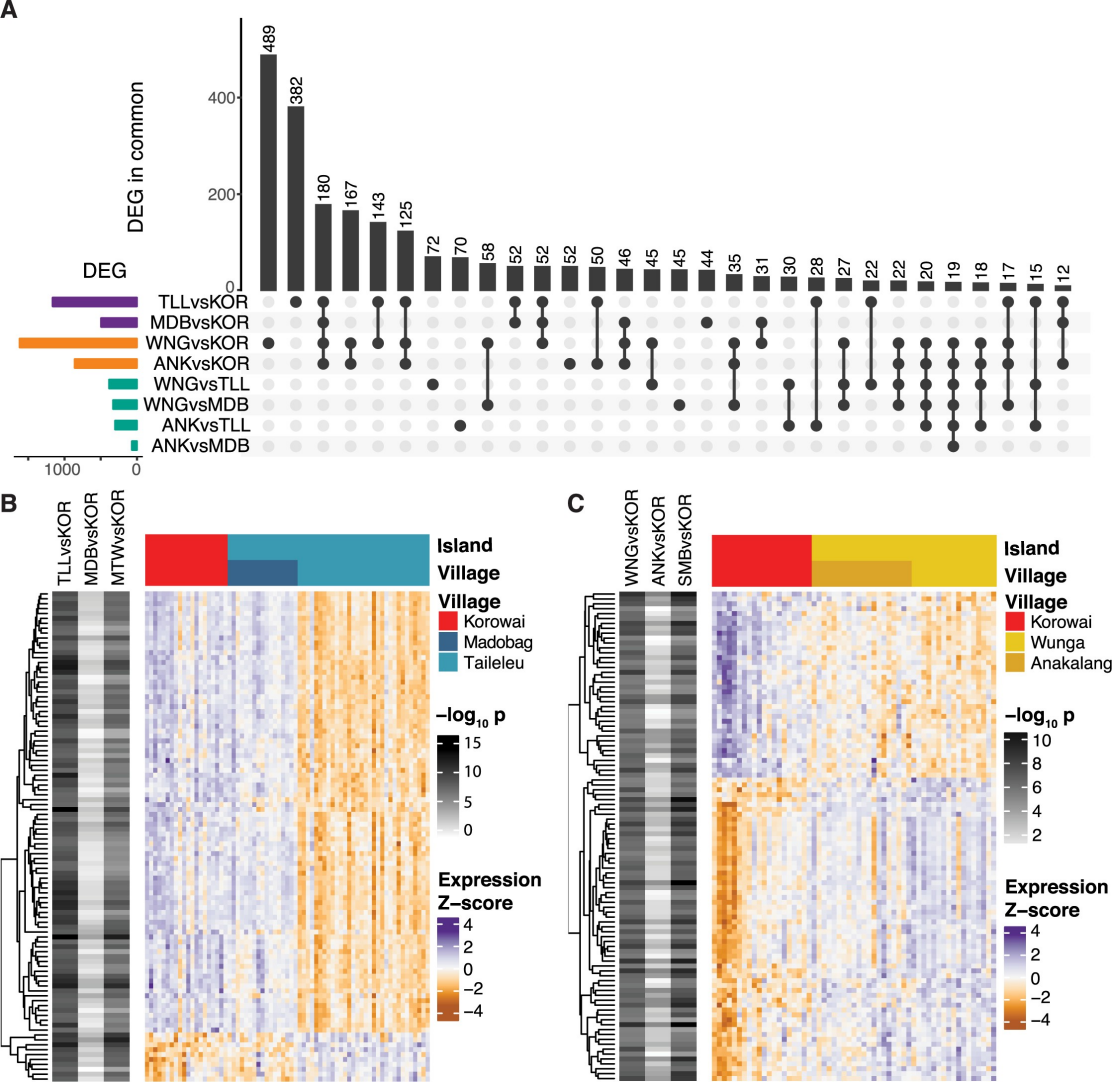
Differential gene expression trends at the village level partially reflect inter-island trends. **(**A) Sharing of village-level DEG signal across all possible inter-island contrasts. (B) Top 100 DEGs between Taileleu and the Korowai that are not DE between Madobag and the Korowai. (C) Top 100 DEGs between Wunga and the Korowai that are not DE between Anakalang and the Korowai.

We then repeated our differential expression and methylation analyses between villages. At a log_2_ FC threshold of 0.5 and an FDR of 1%, we are able to recapitulate the main findings of our island-level analyses, although additional trends emerge (Figs 4 and S9). Detectable differences between villages in the same island are small, with only 62 DMPs and 55 DEGs between the two Mentawai villages of Madobag and Taileleu, and 23 DMPs and 1 DEG, *IDO1* (a modulator of T-cell behavior and marker of immune activity [56]; *p* = 0.009, log_2_ FC = -1.49), between the Sumbanese villages of Wunga and Anakalang, echoing their limited separation in PCA. Similarly, we find low numbers of DEGs and DMPs across all comparisons involving Sumba and Mentawai (Fig 4), again recapitulating the observations we made at the island level (Fig 2). Overall, there appears to be high concordance between genes identified as DE at the island and village level (S10 Fig), with a high degree of correlation between village- and island-level results, as expected (S15 Table). However, when comparing villages between islands, we identified substantially more DMPs and DEGs between Taileleu and Korowai (14,231 and 1,143, respectively) than between Madobag and Korowai (9,787 and 484, respectively), although both Taileleu and Madobag are located in Mentawai and have very similar genetic backgrounds. Similarly, we identified more DMPs and DEGs between Wunga and Korowai (31,905 and 1,592, respectively) than between Anakalang and Korowai (26,317 and 843, respectively).

To understand why we may observe these patterns, we focused on genes that exhibit discordant patterns between the villages on a single island. DEGs between Taileleu and Korowai, but not between Madobag and Korowai (Fig 4B), tend to have similar expression profiles in Madobag and Korowai, whereas DEGs between Wunga and Korowai but not between Anakalang and Korowai (Fig 4C) seem to be expressed at an intermediate level in Anakalang. These differences are not correlated with known technical confounders such as differences in RNA quality or in variability within villages (S11 Fig). Indeed, their presence in both the DNA methylation and RNA sequencing results argues against sample processing artifacts. In order to confirm that these patterns were not driven by differences in sample size, we randomly subsampled each village to 10 individuals and repeated DEG testing 10^3^ times. There are consistently more DEGs between Wunga and Korowai than Anakalang and Korowai (t-test *p* < 10^−30^) as well as between Taileleu and Korowai than between Madobag and Korowai (*p* < 10^−30^). Given the genetic homogeneity we observe within islands (S5 and S8 Figs), we reasoned that these observations may be driven by interactions between genetics and differences in the fine-scale local environment at each sampling site, although a comparison of rainfall and mean monthly temperatures across all five sites did not support these factors as drivers (S12 Fig). While there are clear differences between islands across all climate variables we have considered, climate is generally homogeneous within islands, and thus is unlikely to be responsible for the trends we observe. On the whole, our results highlight the importance of detailed data collection and thorough sampling from regions spanning diverse genomic and environmental clines, if we are to elucidate gene-by-environment interactions.

## Discussion

Although Island Southeast Asia accounts for nearly 6% of the world's population and contains substantial ethnic and genetic diversity [13], genomic characterisation of this region lags drastically behind other regions of the world. The first regional large-scale set of publicly available human whole genome sequences was published in 2019 [6]; to our knowledge, there is only one study of gene expression from the region, of patients with malaria from the northern tip of Sulawesi [7]. In contrast, our work represents the first characterization of gene expression and DNA methylation levels across self-reported healthy individuals from geographically and genetically distinct populations in Indonesia, and more broadly from Island Southeast Asia. We have surveyed three sites with genetically distinct populations, spanning the Asian/Papuan genetic cline that characterises human diversity in the region, and we also sampled multiple villages in two of the islands (Sumba and Mentawai). Our study design purposefully allows us to explore both genetic (primarily between islands) and environmental (both between and within islands) contributions to expression and methylation differences, a result that is further highlighted in our inter-village analysis, where we observe some small-scale village-specific effects (Fig 4).

Indeed, while we find differentially expressed genes and differentially methylated CpGs in most location comparisons (Fig 2), the most numerous, reproducible and largest effect changes were found when comparing either the Sumbanese or Mentawai with the Korowai. Many of these results feature genes involved in immune function, suggesting a potentially adaptive response to local environmental pressures. For example, beyond consistent enrichment for immune-associated GO and KEGG terms, the top 20 strongest DEG signals between the Mentawai and the Korowai include genes involved in antigen presentation in both innate and adaptive immune cells (*MARCO* and *SIGLEC7*, respectively; *MARCO p* = 3.7×10^−13^; *SIGLEC7 p* = 1.1×10^−12^; these genes are also differentially expressed between Sumbanese and the Korowai (*MARCO p* = 1.1×10^−9^; *SIGLEC7 p* = 1.2×10^−11^; S13 Fig). Polymorphisms within MARCO, which is expressed on the surface of macrophages, have been repeatedly shown to associate with susceptibility of infection by *Mycobacterium tuberculosis* and *Streptococcus pneumoniae* in multiple populations worldwide [57–60]; some of these variants have been subsequently shown to have a direct impact on antigen-binding [61]. Our MethylMix analyses identify differences in SIGLEC7 expression as being potentially driven, at least in part, by methylation differences in its promoter region (Fig 3B).

Although we have generated a preliminary set of genotype calls from our RNA-sequencing data, in the absence of whole-genome-level results from our samples, it is challenging to identify whether these signals are also associated with selective signals or driven entirely by environmental differences; neither of these genes has been identified in previous scans of Denisovan introgressions and our current genotype calls do not have sufficient resolution to enable us to directly call introgression in these samples. However, both we and others have previously shown that introgressed Denisovan tracts on the island of New Guinea are enriched for immune genes [6,62], similar to the contributions of Neanderthals to non-African genomes [63,64]. Indeed, our data suggest that Denisovan introgression in New Guinea may be impacting gene expression levels in the Korowai. More broadly, immune challenges have exerted some of the strongest selective forces on humans throughout our species’ history [11]; transmissible diseases endemic in Indonesia range from malaria (both *P*. *falciparum* and *P*. *vivax*) [8] to infections by multiple helminth species and other understudied tropical diseases [2]. Tuberculosis remains a major health concern in the region, with the World Health Organisation reporting nearly half a million new cases in 2017 [65].

Others have sought to characterise the interplay between genetic and environmental contributions to either expression or methylation levels across limited geographic scales. A study of approximately 1,000 individuals drawn from a founder population in Quebec demonstrated that gene-by-environment interactions–specifically, with air pollution levels–drastically impacted measurements of gene expression in blood, overpowering the effects of genetic relatedness [66]. Equivalent high-resolution Indonesian data are unavailable, and our attempts to associate differences in expression or methylation across small geographic scales by using WorldClim data were inconclusive. Unfortunately, it remains difficult to characterize granular levels of regional heterogeneity in disease burden and infection type, yet our results suggest pressures shaping immune response in Indonesia vary at the local level.

A different study of DNA methylation across rainforest hunter-gatherer and farmer populations in Central Africa showed that methylation captures both population history and current lifestyle practices. However, these two factors impact non-overlapping sets of genes, with differences at immune genes associated with a group’s present-day habitat as well as genomic signals of past positive selection [55]. We observe similar trends here; the Korowai occupy an ecological niche akin to that of African rainforest hunter-gatherers, whereas the inhabitants of Sumba and Mentawai are village-based agriculturalists. Sumba, in particular, is host to a network of traditional communities derived largely from pre-existing Papuans, who first arrived on the island ~50,000 years ago, and incoming Asian farming cultures, that reached the island ~4,000 years ago [14]. Today, Sumba retains a low population density and little contact between villages, as reflected in its extensive linguistic diversity [67]. This has resulted in small, isolated populations of a few hundred to a few thousand individuals that can be identified genetically between villages roughly 10 km apart [14], making it a near unique study system for examining gene-by-environment interactions.

As we move further into the age of personalised and genomic medicine, understanding how genetics and other molecular phenotypes drive disease risk across diverse populations is of crucial importance to ensure benefits are equitably distributed. Already there has been a dramatic expansion of genomic-based tests that are being deployed to identify the risk of disease. However, these tests are largely built using European cohorts and have proven difficult to translate to non-European populations [68–70]. Even within homogeneous populations, environmental factors can have marked effects on gene expression measurements, and on the interpretability of genomic-based tests of disease risk [71], highlighting a secondary risk of such biased European sampling: limiting not only the genomic diversity under study, but the environmental diversity as well, to general detriment. This study provides a valuable first step in the characterization of the processes shaping gene expression changes in Island Southeast Asia.

## Supporting information

S1 Table Sample metadata. (XLSX)

S2 Table Sample sequencing information. (XLSX)

S3 Table Pairwise correlations between all blood deconvolution methods. (XLSX)

S4 Table Summary of DEG/DMP/DMR testing at various thresholds. (XLSX)

S5 Table List of genes with expression levels significantly associated to individual samples' Papuan ancestry proportions. (XLSX)

S6 Table List of probes with methylation levels significantly associated to individual samples' Papuan ancestry proportions. (XLSX)

S7 Table GO enrichment testing results for DEGs. (XLSX)

S8 Table KEGG enrichment testing results for DEGs. (XLSX)

S9 Table GO enrichment testing results for DMPs. (XLSX)

S10 Table KEGG enrichment testing results for DMPs. (XLSX)

S11 Table EWAS catalogue enrichment testing results for DMPs. (XLSX)

S12 Table List of significant MethylMix clusters. (XLSX)

S13 Table KEGG enrichment testing for MethylMix-associated genes. (XLSX)

S14 Table ANOVA on PCA and covariates. (XLSX)

S15 Table Spearman correlation between village and island level across both DEG and DMP tests. (XLSX)

S1 Fig Clustering of the gene expression data before and after batch correction. (TIF)

S2 Fig Distribution of Spearman's pairwise correlation (ρ) values across all levels of the RNA-sequencing data. (EPS)

S3 Fig Clustering of the DNA methylation data before and after batch correction. (TIFF)

S4 Fig Distribution of Spearman's pairwise correlation (ρ) values across all levels of the DNA methylation data. (EPS)

S5 Fig Predicted ancestry proportions for all samples in the data on the basis at different multiple values of K, estimated by ADMIXTURE. (TIFF)

S6 Fig Principal component analyses of our samples relative to published Island Southeast Asia data. (TIFF)

S7 Fig Relationship between the log_2_(FC) of probes and genes across island-level comparisons, as in Fig 2. (A) and (C), DNA methylation probes; (B) and (D), gene expression.
(TIFF)

S8 Fig PCA plots of DNA methylation and gene expression as in Fig 1, with villages and islands indicated. (TIFF)

S9 Fig Sharing of village-level DMP signal across all possible inter-island contrasts. (EPS)

S10 Fig Sharing of DE signals at the island and village levels. (EPS)

S11 Fig Distribution of coefficients of variation (CoV) across villages. (EPS)

S12 Fig Monthly climate fluctuations across the five main village sampling sites. (EPS)

S13 Fig **log_2_ CPM values across all samples for (A) MARCO and (B) SIGLEC7**.
(EPS)

S1 Text Further details on blood deconvolution. (DOCX)
