# Supplementary figures and images for: Genome-wide DNA methylation and gene expression patterns reflect genetic ancestry and environmental differences across the Indonesian archipelago
Source: PLoS Genet. 2020 May 26;16(5):e1008749. doi: 10.1371/journal.pgen.1008749 (PMC7274483; doi:10.1371/journal.pgen.1008749)

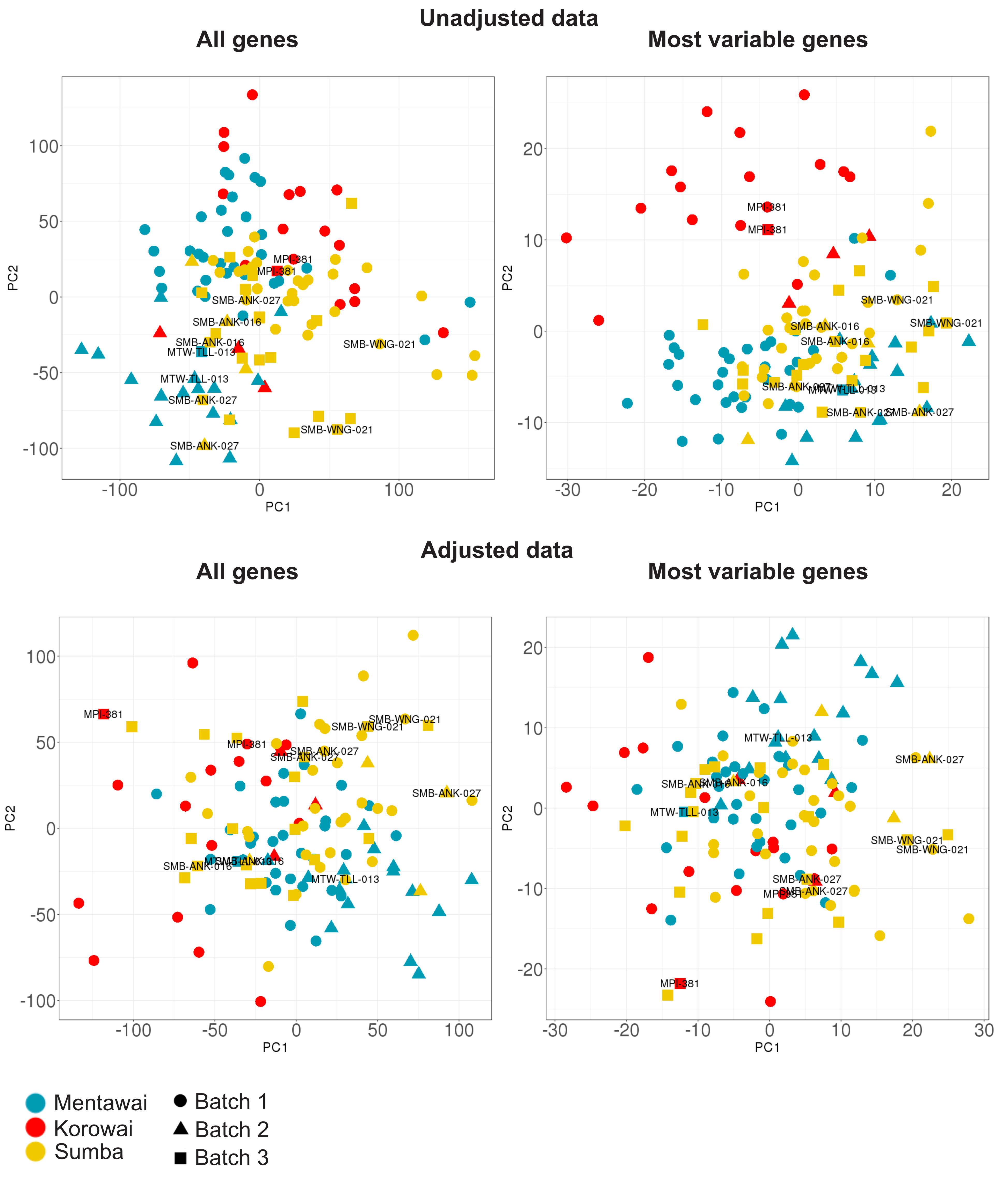

Supplement: S1 Fig — (TIF) [file pgen.1008749.s016.tif]

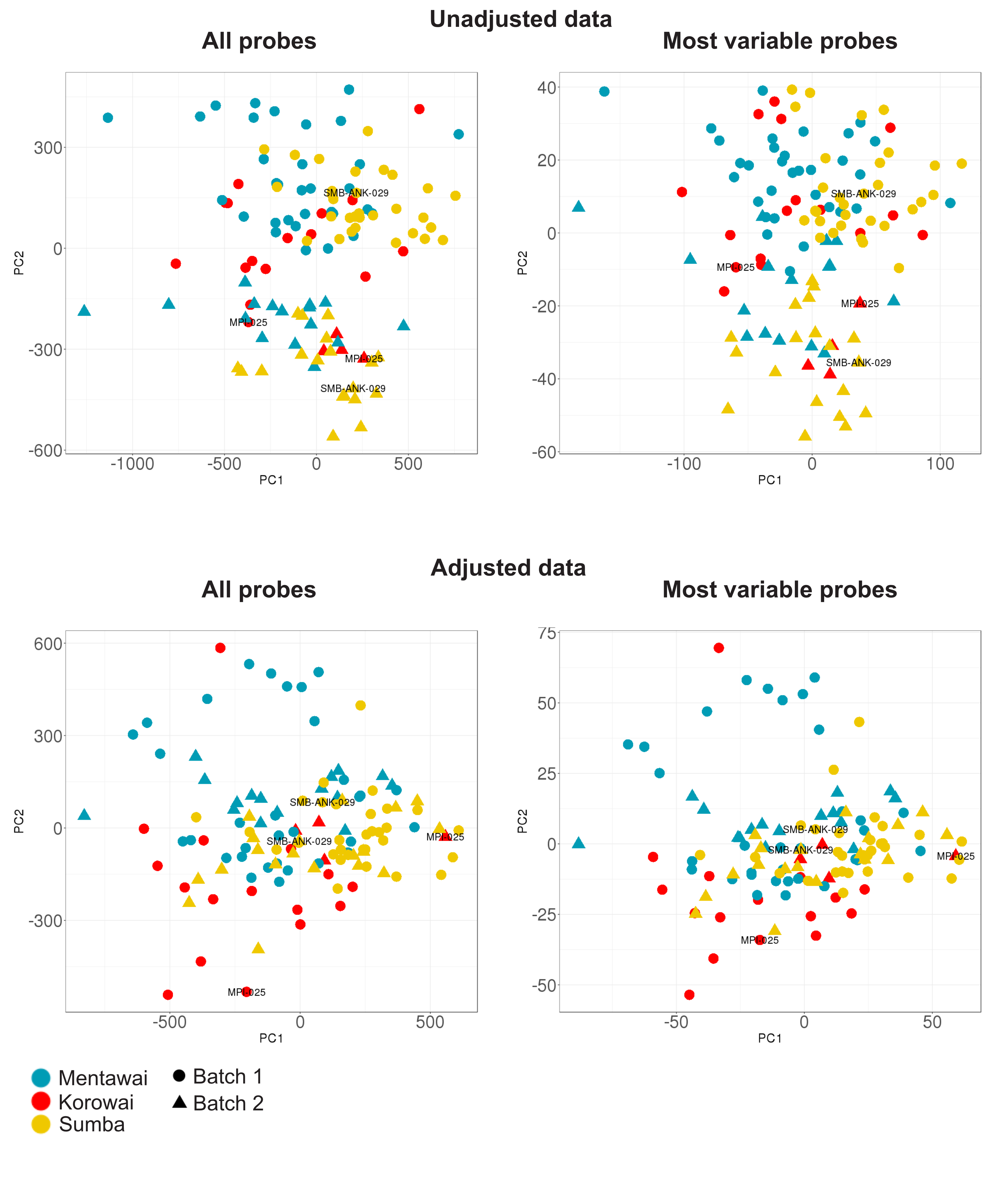

Supplement: S3 Fig — (TIFF) [file pgen.1008749.s018.tiff]

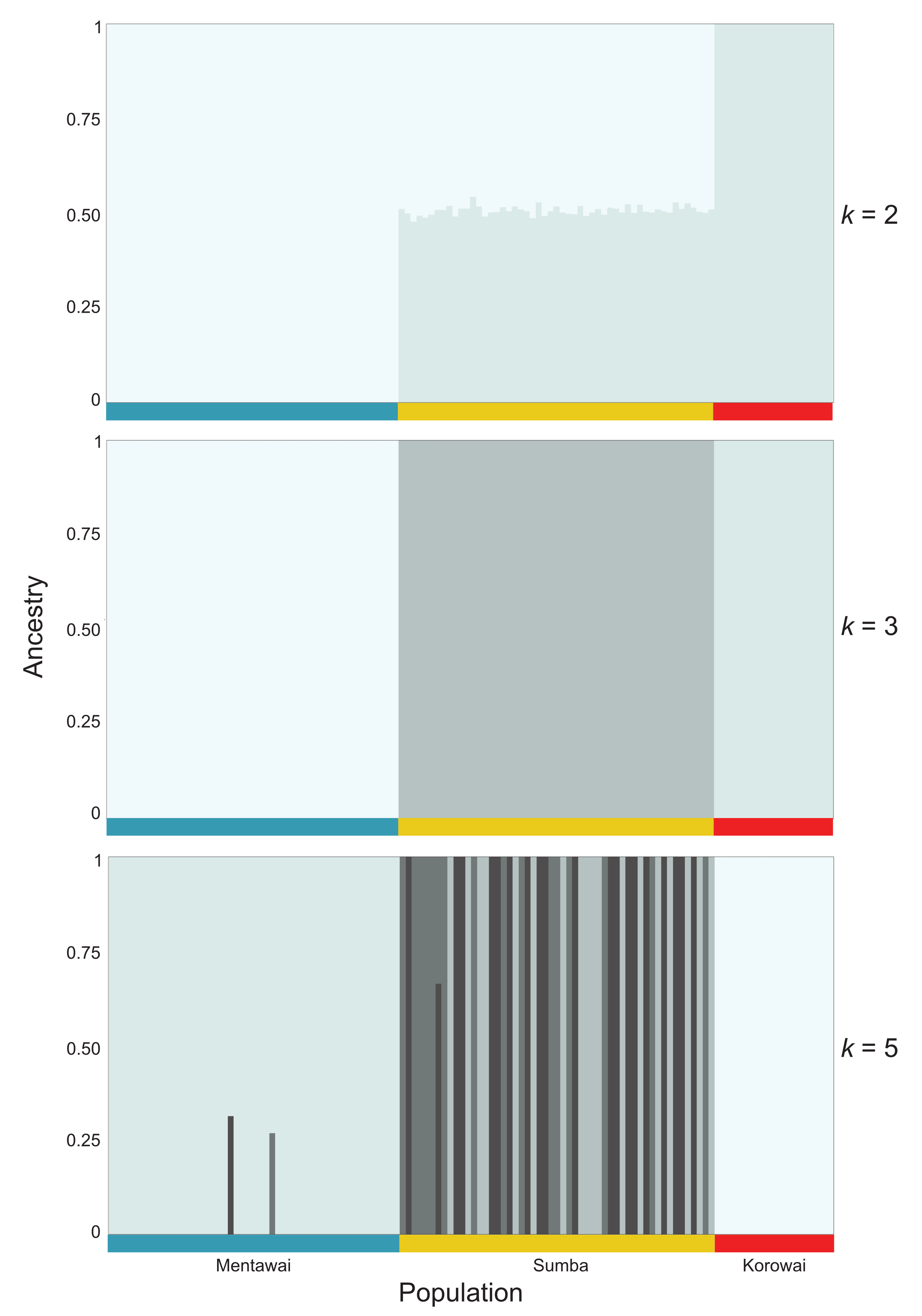

Supplement: S5 Fig — (TIFF) [file pgen.1008749.s020.tiff]

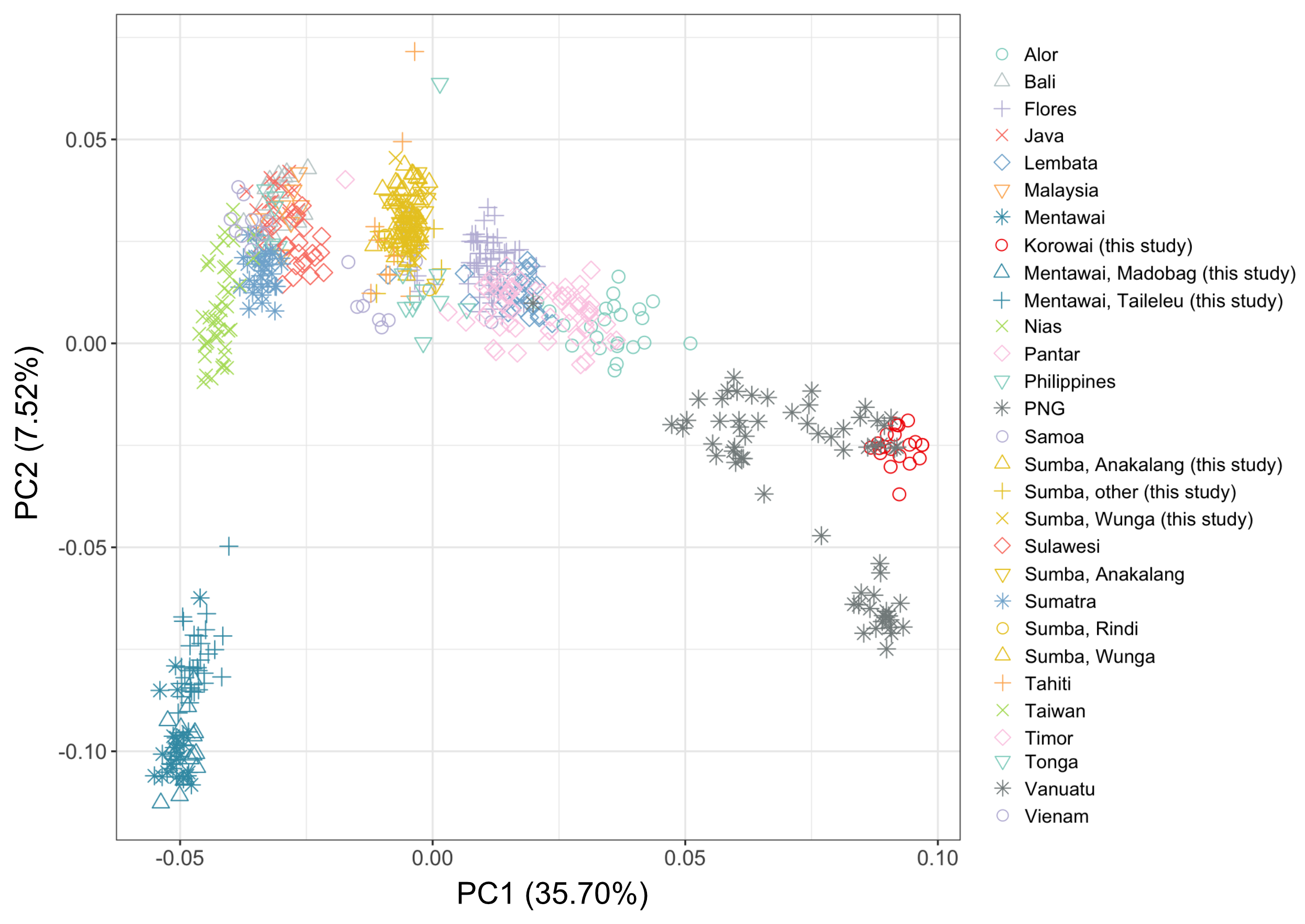

Supplement: S6 Fig — (TIFF) [file pgen.1008749.s021.tiff]

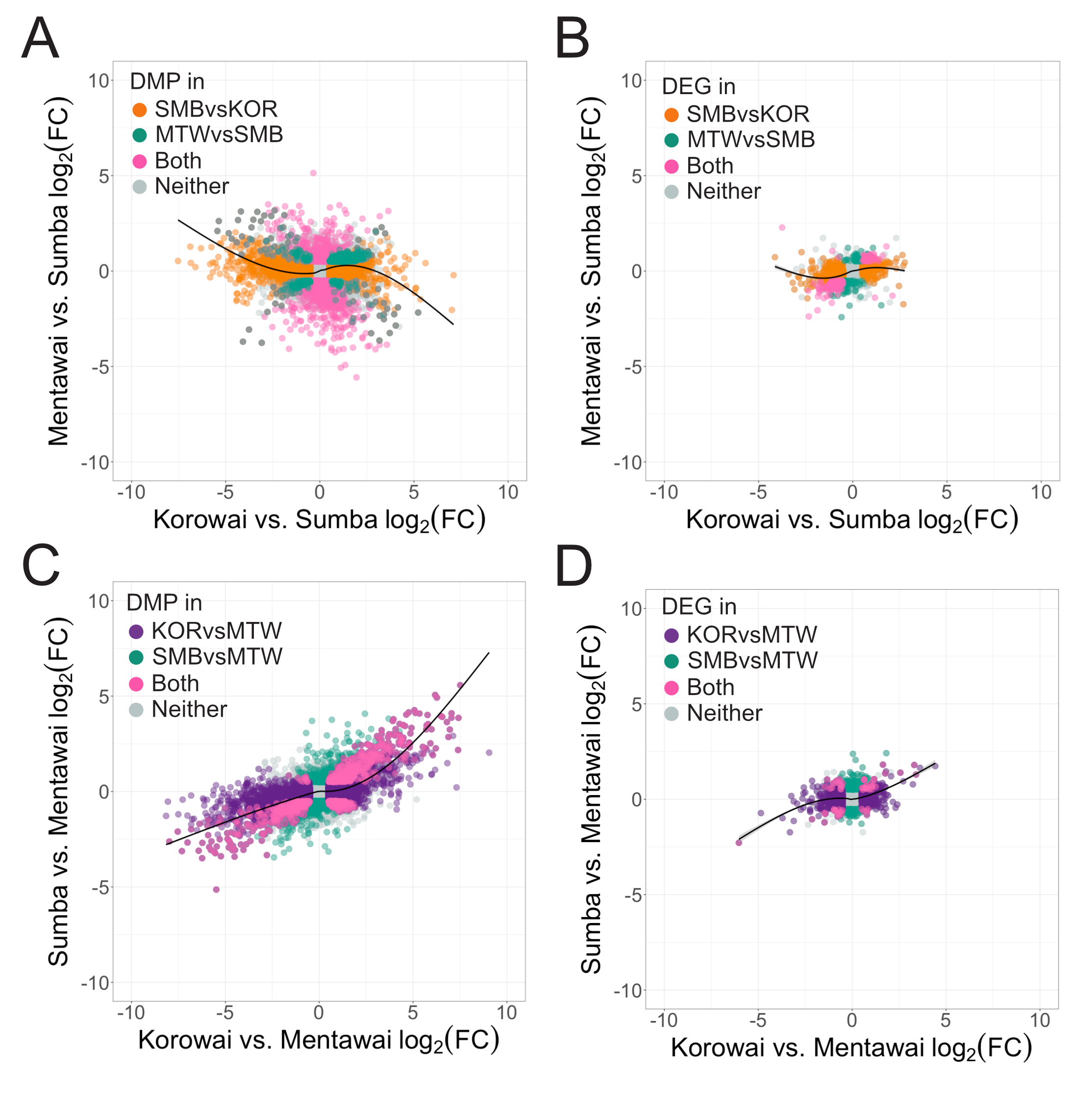

Supplement: S7 Fig — (A) and (C), DNA methylation probes; (B) and (D), gene expression. (TIFF) [file pgen.1008749.s022.tiff]

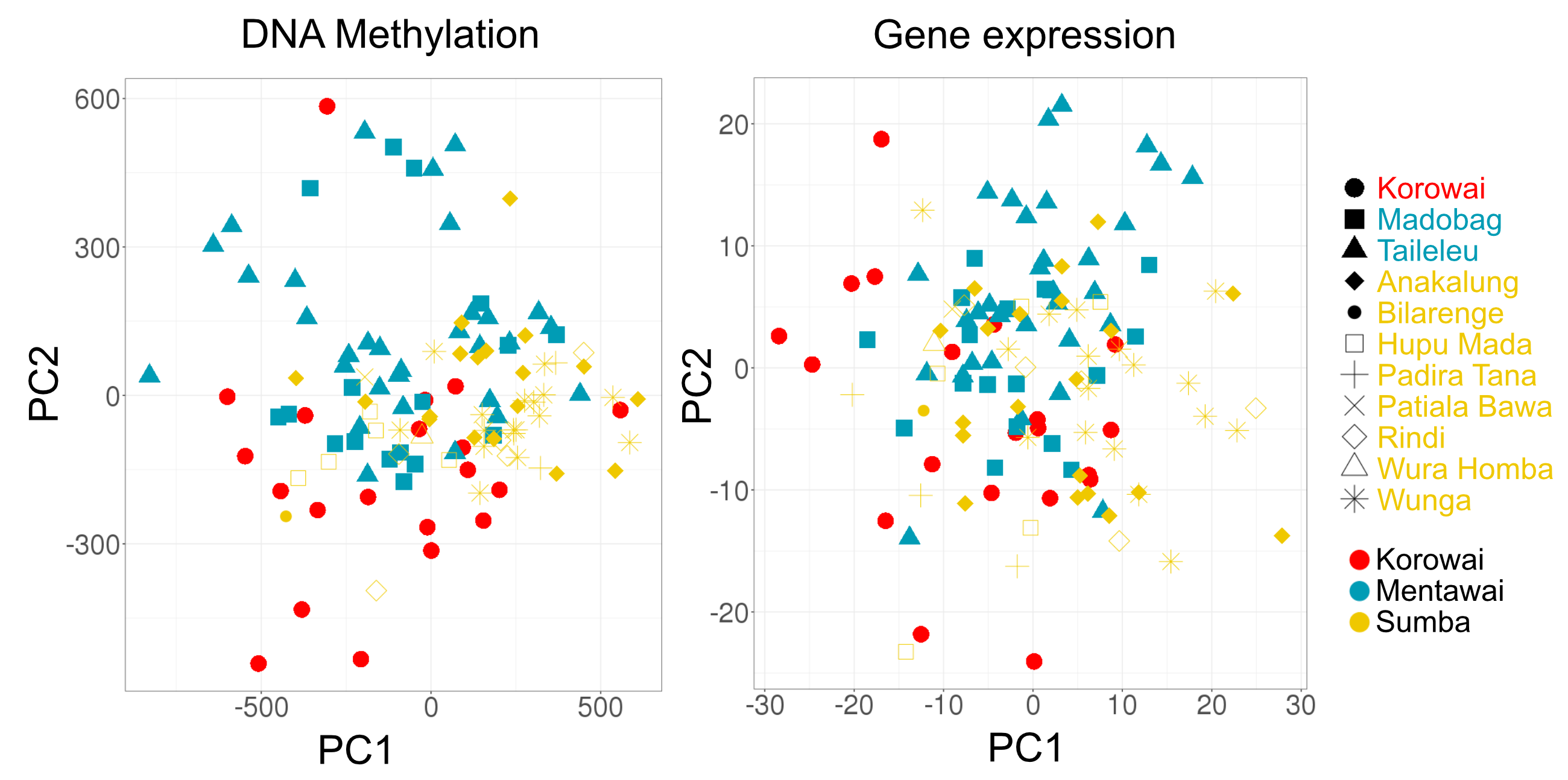

Supplement: S8 Fig — (TIFF) [file pgen.1008749.s023.tiff]
