# Supplementary material for: Genome-wide DNA methylation and gene expression patterns reflect genetic ancestry and environmental differences across the Indonesian archipelago
Source: PLoS Genet. 2020 May 26;16(5):e1008749. doi: 10.1371/journal.pgen.1008749 (PMC7274483; doi:10.1371/journal.pgen.1008749)
Supplement: S1 Text — (DOCX) [file pgen.1008749.s029.docx]

Testing of possible covariates against the first 10 principal components (PC) in the expression data identified estimated blood cell type proportions that are significantly associated with at least one PC. Thus, as described in the main text, these were included as covariates in our differential expression and differential methylation testing models.

In addition, we carried out ANOVAs for all blood cell type abundance estimates from DeconCell (Fig 1 in S1 Text). Three cell types – NK cells, monocytes, and CD4+ T-cells – do differ significantly by village (Bonferroni-corrected ANOVA p = 2.3x10^-5^, 2.9x10^-4^ and 0.024, respectively). Tukey's HSD revealed that in the case of NK cells, these differences were predominantly driven by the Korowai, who had consistently lower NK cell estimates than individuals from Anakalang, Taileleu and Madobag (p = 1.2x10^-5^, 4.9x10^-4^ and 3.8x10^-5^ respectively), but not Wunga (p = 0.054). We also find that individuals sampled in Madobag had significantly lower estimated monocyte counts than people from Anakalang or the Korowai (p = 2.91x10^-5^ and 0.013 respectively) and also observe a difference between Anakalang and Taileleu (p = 0.011). Finally, CD4+ T-cell estimates were significantly lower in Anakalang than in Taileleu and Madobag (p = 0.027 and 0.030 respectively).

We note that mean estimates for all cell types in all villages fall within the range observed in healthy individuals for the DeconCell predictions, and differ little between populations, with significance in our results likely to be driven by sample size.


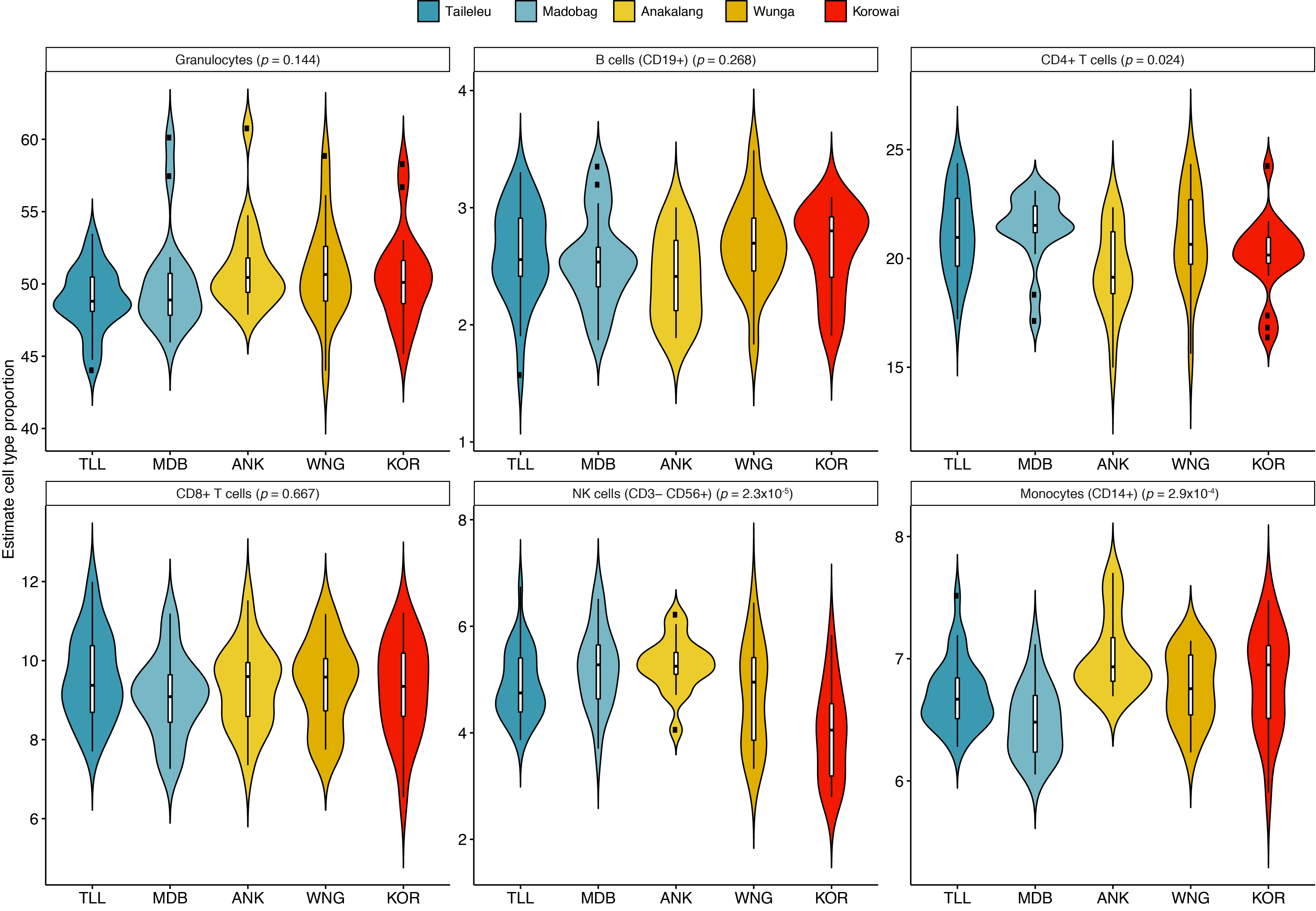


Table 1: Tukey's HSD results, NK cells (ANOVA p = 2.3x10^-5^)

|  | **Mean difference** | **Lower** | **Upper** | **Adjusted *p* value** |
| --- | --- | --- | --- | --- |
| MDB-ANK | -0.069 | -0.785 | 0.647 | 0.999 |
| KOR-ANK | -1.298 | -1.995 | -0.601 | 1.22x10^-5^ |
| TLL-ANK | -0.371 | -1.001 | 0.259 | 0.477 |
| WNG-ANK | -0.597 | -1.324 | 0.130 | 0.160 |
| KOR-MDB | -1.229 | -1.926 | -0.532 | 3.76x10^-5^ |
| TLL-MDB | -0.302 | -0.932 | 0.328 | 0.671 |
| WNG-MDB | -0.528 | -1.255 | 0.199 | 0.265 |
| TLL-KOR | 0.927 | 0.318 | 1.535 | 4.94x10^-4^ |
| WNG-KOR | 0.701 | -0.007 | 1.409 | 0.054 |
| WNG-TLL | -0.226 | -0.868 | 0.417 | 0.865 |

Table 2: Tukey's HSD results, monocytes (ANOVA p = 2.9x10^-4^)

|  | **Mean difference** | **Lower** | **Upper** | **Adjusted *p* value** |
| --- | --- | --- | --- | --- |
| MDB-ANK | -0.539 | -0.841 | -0.237 | 2.91x10^-5^ |
| KOR-ANK | -0.195 | -0.488 | 0.099 | 0.357 |
| TLL-ANK | -0.317 | -0.582 | -0.051 | 0.011 |
| WNG-ANK | -0.256 | -0.562 | 0.051 | 0.148 |
| KOR-MDB | 0.345 | 0.051 | 0.639 | 0.013 |
| TLL-MDB | 0.223 | -0.043 | 0.488 | 0.145 |
| WNG-MDB | 0.284 | -0.023 | 0.590 | 0.084 |
| TLL-KOR | -0.122 | -0.379 | 0.134 | 0.676 |
| WNG-KOR | -0.061 | -0.360 | 0.237 | 0.979 |
| WNG-TLL | 0.061 | -0.210 | 0.332 | 0.971 |

Table 3: Tukey's HSD results, CD4+ T-cells (ANOVA p = 0.024)

|  | **Mean difference** | **Lower** | **Upper** | **Adjusted *p* value** |
| --- | --- | --- | --- | --- |
| MDB-ANK | 1.959 | 0.130 | 3.788 | 0.030 |
| KOR-ANK | 0.661 | -1.119 | 2.441 | 0.839 |
| TLL-ANK | 1.745 | 0.135 | 3.354 | 0.027 |
| WNG-ANK | 1.480 | -0.378 | 3.337 | 0.183 |
| KOR-MDB | -1.297 | -3.077 | 0.483 | 0.261 |
| TLL-MDB | -0.214 | -1.823 | 1.395 | 0.996 |
| WNG-MDB | -0.479 | -2.336 | 1.378 | 0.952 |
| TLL-KOR | 1.083 | -0.470 | 2.637 | 0.304 |
| WNG-KOR | 0.818 | -0.991 | 2.627 | 0.718 |
| WNG-TLL | -0.265 | -1.906 | 1.376 | 0.991 |

Abbreviations:

ANK – Anakalang (Sumba Island)

KOR – Korowai (New Guinea Island)

MDB – Madobag (Mentawai Island)

TLL – Taileleu (Mentawai Island)

WNG – Wunga (Sumba Island)
